# Machine learning for MEG during speech tasks

**DOI:** 10.1038/s41598-019-38612-9

**Published:** 2019-02-07

**Authors:** Demetres Kostas, Elizabeth W. Pang, Frank Rudzicz

**Affiliations:** 1 University of Toronto, Toronto, Canada; 2 Vector Institute for Artificial Intelligence, Toronto, Canada; 3 Hospital for Sick Children, Toronto, Canada; 4 SickKids Research Institute, Toronto, Canada; 5 Li Ka Shing Knowledge Institute at St Michael’s Hospital, Toronto, Canada; 6 Surgical Safety Technologies Inc, Toronto, Canada

## Abstract

We consider whether a deep neural network trained with raw MEG data can be used to predict the age of children performing a verb-generation task, a monosyllable speech-elicitation task, and a multi-syllabic speech-elicitation task. Furthermore, we argue that the network makes predictions on the grounds of differences in speech development. Previous work has explored taking ‘deep’ neural networks (DNNs) designed for, or trained with, images to classify encephalographic recordings with some success, but this does little to acknowledge the structure of these data. Simple neural networks have been used extensively to classify data expressed as features, but require extensive feature engineering and pre-processing. We present novel DNNs trained using raw magnetoencephalography (MEG) and electroencephalography (EEG) recordings that mimic the feature-engineering pipeline. We highlight criteria the networks use, including relative weighting of channels and preferred spectro-temporal characteristics of re-weighted channels. Our data feature 92 subjects aged 4–18, recorded using a 151-channel MEG system. Our proposed model scores over 95% mean cross-validation accuracy distinguishing above and below 10 years of age in single trials of un-seen subjects, and can classify publicly available EEG with state-of-the-art accuracy.

## Introduction

Speech development, from infancy to adulthood, is a remarkable and uniquely human process. Language and speech in the adult brain spans a diverse set of regions and interconnections from brain stem to cerebral cortex^1–3^, but higher level language abilities are typically left-hemisphere lateralized for approximately 90% of the general population, although this can range from 70% to 95% in populations with distinct left and right handedness respectively^1,4,5^. Children develop this lateralization over time, with young children typically showing bilateral representation of core language areas in young childhood, with increasing leftward lateralization in the pre-teen and teen years^4,6^. A common experimental paradigm for demonstrating this is the verb-generation task, which requires a subject to produce a verb that is associated with an object/noun with which they have been provided (presented for example, using an image)^4^. Speech articulation divorced from language is much less lateralized in comparison to *higher level* language faculties regardless of age^1^, and in terms of cortical systems, heavily engages the Rolandic cortex to integrate somatosensory and motor control representations to command the speech articulators^1^. Although this process is less lateralized than higher level language function, non-word monosyllabic, and multisyllabic experiments still show some left-lateralization^7^. We consider a dataset made up of a combination of a verb-generation task, the monosyllabic utterance /*pah*/ and the multisyllable non-word /*pah tah kah*/ with overlapping sets of subjects recorded using magnetoencephalography (MEG), to demonstrate age-related language and speech development.

Machine learning (ML) has been used in brain computer interfaces (BCIs), including for silent speech (imagined or covert)^8–10^. Typically, data are collected and pre-processed using a variety of techniques, such as cropping, trial averaging, normalization, band-pass filtering, and spatial transforms such as principle component analysis (PCA), independent component analysis (ICA) and common spatial patterns (CSP)^11,12^. The goal of the preprocessing stage is to overcome the low signal-to-noise ratios typical of these recordings. After these initial steps, features are calculated, often representing spectral characteristics in the canonical brain frequency bands (*δ*, *θ*, *α*, etc.), and summary statistics.

An advantage of this approach is that expert knowledge about the data can help circumvent relatively poor signal quality and encourage ML models to distinguish between classes using established correlates. However, this comes with potential bias of expert knowledge, and the underlying assumptions it may reinforce. In contrast, ‘deep’ ML models can achieve many of the stages of the pipeline intrinsically, and consider potentially unknown correlates. We propose two new DNN architectures, the first labelled SCNN is a mainly convolutional neural network (CNN), and the second labelled Ra-SCNN is an augmented SCNN network that employs a recurrent layer and attention mechanism. Both networks are trained with raw single trials, that exploit the pipeline typically used for BCIs. Here they are trained to predict the age of normally developing (in terms of speech abilities) children aged 4–18, performing at least two of three tasks commonly used to evaluate speech and language development. They are evaluated with single trials of subjects unseen during training. This training and evaluation context is established to encourage the DNNs to make predictions on the grounds of detected differences in the development of speech and/or language.

**Figure 1:**
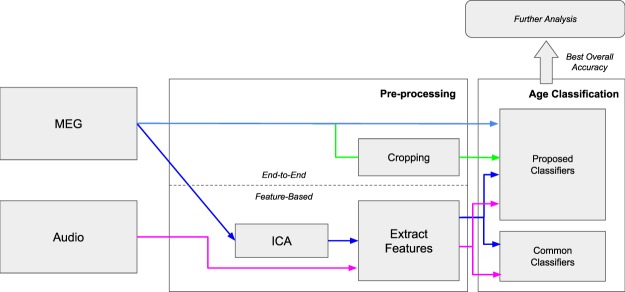
Diagram of the different processing steps performed for each dataset to compare the feature-based and end-to-end approaches. MEG and audio recordings are used with the feature-based approach to benchmark the end-end-end models using raw MEG. The best end-to-end model is then interrogated to reveal how decisions are made. Each colour represents a dataset configuration that is produced.

We compare the performance of a wide-range of classifiers, including our proposed models, when trained with features extracted from the MEG recordings, and as reference the audio recordings, of each trial. We herein refer to this process as a *feature*-*based* approach. This is compared to our proposed DNNs trained with the same MEG recordings that have been minimally processed, and optionally augmented in number through cropping, which we call an *end*-*to*-*end* approach. The best performing *end*-*to*-*end* model is then thoroughly interrogated: we evaluate the learned weights’ carry-over performance predicting which of the three speech tasks is being performed, we use activation maximization to examine the most successful network’s inner workings, and finally, we demonstrate that our models are a suitable classifier for encephalographic data more generally, by comparing the performance of our DNN architectures against the state-of-the-art for a public dataset of EEG recordings used in BCI applications. The steps are outlined in Fig. 1.

## Results

### Age classification

Table 1 shows a convincing difference between feature-based and end-to-end approaches. The end-to-end approaches all scored nearly 90% or higher mean five-fold cross validation accuracy. Analysis with a Friedman test considering performance of all folds clearly indicated real differences with ${\chi }_{13}^{2}=52.789$ and $p < {10}^{-6}$. All end-to-end models significantly outperformed logistic regression trained with both audio and MEG features ($p < 0.05$ after single-step correction), and the SCNN trained with raw un-augmented data also significantly outperformed the FFNN trained with the reduced MEG features dataset. Although this was not a unanimous demonstration that the end-to-end models were superior, we saw this as a fairly promising indication of performance. Of particular interest are the SCNN models trained without crop augmentation and the Ra-SCNN model with crop augmentation.

**Table 1:** Classification accuracy of *age* >= 10 versus *age* < 10.

Technique	Dataset	Model	Mean %	Dev. %
Feature-Based	Audio	Logistic Regression	54.3	6.44
		Linear SVM	73.9	3.45
		FFNN	71.7	2.44
		SCNN	65.9	9.35
		Ra-SCNN	67.5	13.9
	MEG	Logistic Regression	63.5	5.64
		Linear SVM	69.2	5.74
		FFNN	66.3	4.93
		SCNN	69.7	7.03
		Ra-SCNN	72.3	1.74
End-to-End	MEG	SCNN	95.1	2.31
		Ra-SCNN	92.6	5.79
	MEG (+crop. aug.)	SCNN	89.2	7.93
		Ra-SCNN	93.4	0.93

We compared standard feature classifiers of logistic regression, linear kernel support vector machine (Linear SVM), and a fully-connected feed-forward neural network (FFNN) to our proposed models trained end-to-end. The end-to-end models were also trained with an augmentation strategy that artificially increases the number of training points using cropped sub-sequences of trials.

### Finer-Grained Prediction

**Figure 2:**
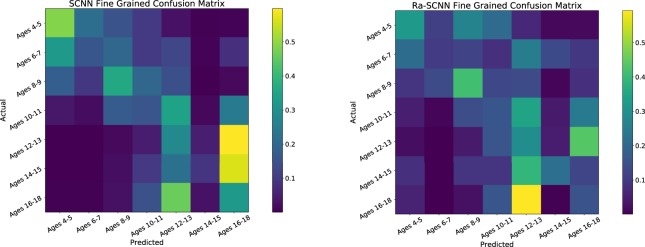
Confusion matrix for both the SCNN (left) and Ra-SCNN (right) fine-grained (seven-way) age classification task. Each square corresponds to the rate of predicted class (columns) for each actual class (rows). The SCNN had noticeably empty bottom left and top right corners, indicating misclassification of very young for very old, and the opposite condition, is very uncommon. This was less pronounced for the Ra-SCNN.

To model the fundamentally continuous nature of the phenomena that these data were meant to represent, we also considered targeting seven age categories, each two years in length (and a final category of three years), rather than the binary targets presented above, using the two highest performing configurations from the previous task (the performance of more configurations are available online in Supplementary Table S1). As might be expected, these did not yield accuracies as high as those from the binary models. Table 2 reports this accuracy, and what we called *one*-*off accuracy*: the percentage of predictions that were correctly predicted, or predicted to be one of the immediately neighbouring classes with respect to the correct class. These are both reported as mean and standard deviation of five-fold cross validation on the same fold divisions and held-out test subjects as the binary task. A Wilcoxon signed-rank test found no significant difference between the SCNN and Ra-SCNN model, but this was somewhat weak with *V*_4,*two*–*sided*_ = 14 and *p* = 0.125. The *one*-*off accuracy* and the diagonal trends exhibited in Fig. 2, particularly the SCNN, suggested that errors were dominated by confusion with nearby age categories, rather than randomly distributed. Interestingly, the SCNN seemed to be split along the age category of 10 and 11 years old, with subjects younger than 10 most often predicted as one of the three categories 11 years old and younger, and rarely predicted as older than 11. Likewise for the subjects older than 11 years of age. They were very rarely predicted to be younger than 10. This served as an indicator that the separation used for the binary task was quite reasonable.

**Table 2:** Classification accuracy of the SCNN and Ra-SCNN models trained to predict seven age categories: 4–5, 6–7, 8–9, 10–11, 12–13, 14–15, 16–18 (both inclusive).

Model	Accuracy		One-off Accuracy	
	Mean %	Dev. %	Mean %	Dev. %
SCNN	28.6	7.91	59.9	10.9
Ra-SCNN (+crop. aug.)	25.0	1.46	50.2	2.36

Both models were trained end-to-end using raw data, and the Ra-SCNN had an augmented set of training points through cropping. *One*-*off Accuracy* denotes the rate of predicting the correct age range or immediately neighbouring age range.

### Task Prediction

With the performances demonstrated above, it is clear that the SCNN and Ra-SCNN architectures showed some promise compared to a more conventional feature-based pipeline; however, it remained to be seen how much of this performance was a result of detected differences in speech development. Here we considered whether the most successful model’s parameters (i.e. the *previously learned* weights from above) applied directly to another speech task independent of age. Starting with an un-trained (randomly initialized) SCNN network of the same configuration used for both age prediction tasks, the network was trained to predict which of the three tasks was being performed. This was compared to a SCNN network that was already trained from the binary prediction task, specifically the SCNN network that achieved the highest single fold accuracy in the binary age classification task. These weights were then fixed, so that the network was used as a fixed feature extractor, and the final layer was replaced with a simple SVM classifier that was trained to classify which task was being performed from the fixed SCNN features.

We see in Table 3 that both of these networks comfortably exceeded the level of chance and the 38.13% majority class threshold (as there was an imbalance of classes, which is addressed during training with a penalized loss). These accuracies were not as impressive as the age prediction task, but they did demonstrate performance that was remarkably similar to each other. A paired Wilcoxon test showed that these distributions were not significantly different, with *V*_4,*two*–*sided*_ = 9.0 and *p* = 0.8125. Therefore, the weights (and thus features) that were learned to accurately perform the age classification task performed equivalently to newly trained weights when predicting a separate speech task independent of age. We saw this as evidence that the features are speech related, rather than expressing characteristics correlated only to age, or some other aspect of the task.

**Table 3:** Accuracy distinguishing between the three speech tasks, across all five folds, using the highest performing SCNN architecture from the binary classification task.

Model Weights	Fold 1	Fold 2	Fold 3	Fold 4	Fold 5	Mean %	Dev. %
Newly Trained	47.1	49.0	48.0	41.7	47.7	46.7	2.88
Previously Trained	48.6	47.1	46.5	47.3	44.7	46.8	1.41

*Newly Trained* represents a network with weights that were randomly initialized, and trained end-to-end to predict the speech task. *Previously Trained* describes the performance of the same architecture, but with weights fixed to the values from the highest performing single fold in the binary classification task. Instead, the model’s final output layer is replaced with a SVM with a linear kernel, which was then trained to predict the task.

### Model Activations

**Figure 3:**
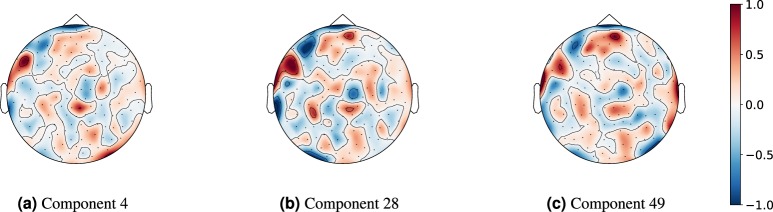
Three examples of input channel relative intensities, interpolated using expected channel locations, that maximized the output of the spatial stage for three different components (kernels) at this stage. Components (**a** and **b**) show a common pattern of spatial mixing, with a particularly strong relative intensity localized around channels near the inferior frontal or perhaps dorsolateral prefrontal regions. This region is fairly consistently represented in many of the components of the spatial stage, as can be seen in Supplementary Table S1. Component (**c**) has had its colour scale reversed to be more similar to (**a** and **b**), as negative weights are ubiquitous throughout any neural network and thus may be flipped downstream. The plots were constructed using bilinear interpolation so that they were mapped across expected channel locations. These plots were made using the MNE python library (available at https://www.martinos.org/mne/stable/index.html).

**Figure 4:**
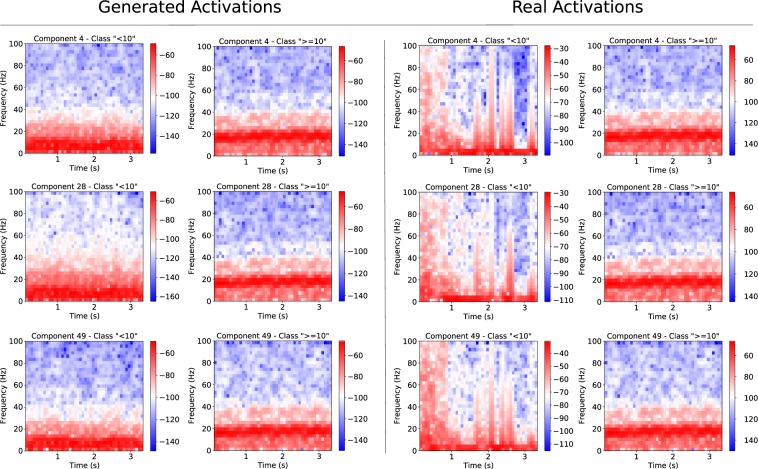
Power spectral density spectrograms in dB scale for activations that maximize output classes. Plots on the left represent generated activations that maximized the prediction of classes <10 and >=10 years old with respect to component 4 (Fig. 3a), component 28 (Fig. 3b) and component 49 (Fig. 3c). Plots on the right show activations from correctly predicted real trials that maximized the prediction of the two output classes. Each row of spectrograms corresponds to activations of components 4, 28 and 49 respectively.

Generating data to maximize the input at some point in the network with respect to an output downstream in the network (both intermediate nodes and final outputs) can provide insight into the preferred characteristics of incoming data and activation patterns within the network. This approach has been used previously to show the characteristics of individual layers and preferred input for image classification networks^13^. We employed input maximization using the model with the highest single fold binary test accuracy to ensure that we generated data that had minimal characteristics that were not pertinent to classifying age. This resulted in using the SCNN model trained with the second fold of the raw MEG dataset. Figure 3 demonstrates the relative intensities of the 151 MEG channels (bilinear interpolation over expected channel locations on scalp image) that maximized three spatial filters at the final spatial summary layer. Correspondingly, Fig. 4 shows the spectral content of generated activations that maximized these same spatial features with respect to the two output classes, juxtaposed against the activations of these same filters from accurately and highly confidently predicted real trials.

Throughout the components, there was a common patch of relative intensity found in approximately the inferior frontal or perhaps dorsolateral prefrontal regions. All three components shown in Fig. 3 demonstrated this to some degree, although component 49 (part (c)) showed this after flipping the colour scale. This inversion was done for easier comparison, as weights further downstream may very well flip the effect of a component like this (likewise for components (a) and (b)). The spectrograms of generated activations were fairly uniform across all components, with spectral density concentrated between approximately 0–14 H*z* in the <10 years old class, and concentration 15–22 H*z* for >=10 years old. All of the generated activations appeared very stationary, and showed no particular emphasis around the event time *t* = 1.5 *s*. The generated activations that maximized the >=10 did however show more activity overall in the high *beta*, low *gamma* rhythms and this is particularly evident in components 4 and 49.

The real activations, unlike their generated counterparts were, unsurprisingly, not as stationary. These also did tend to show a marked change after the *t* = 1.5 *s* mark. However, much like the generated activations, the spectro-temporal differences between the three components are minimal. All components and their generated activations with respect to the two output classes are included in Supplementary Fig. S2.

### Baseline Recordings

The homogeneity of the generated activations above could have been evidence that classification was relying on some non-event related signals; for example, some correlation between resting frequency and age. To consider if this was the case, we again explored the highest-accuracy fold SCNN model from the binary task, and considered how it well it classified baseline recordings for each test subject and each experiment task, consisting of the brief recording of subjects before the sequence of trials for each task. This was compared to the performance of subtracting a simple model of baseline activity, made by averaging the inital recordings across all tasks for each subject, from an accurate test trial for each subject and test condition.

Table 4 suggests that classifying non-task recordings resulted in dramatically lower performance than classifying real trials, and subtracting the mean baseline from test points was not nearly as detrimental. Testing for independence of the *Initial Baseline* and *Test Minus Average Baseline* groups showed a significant difference between the groups with ${\chi }_{2}^{2}=6.256$ and $p < 0.013$.

**Table 4:** Number of correct versus incorrect predictions when considering baseline recordings of held-out test subjects, compared to subtracting average baseline of each subject from correctly predicted, and highly confident test trials with corresponding subject and task.

Predictions	Initial Baseline Alone	Test Minus Average Baseline
Correct	15	23
Incorrect	11	3

### Obscuring Trials

**Figure 5:**
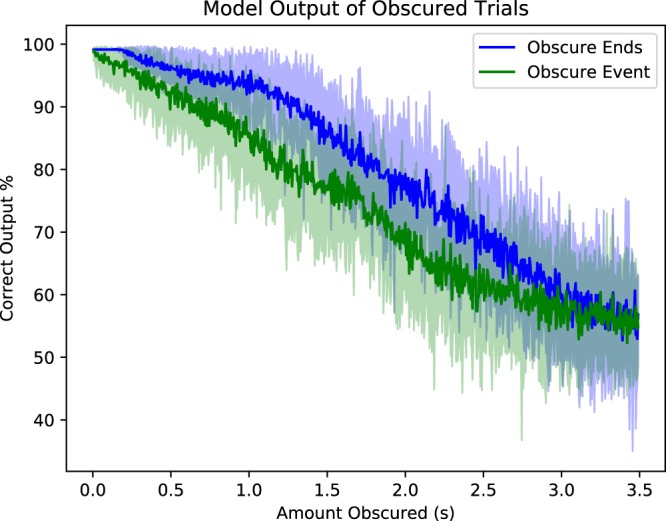
Output intensity of model corresponding to the correct prediction, when obscured by white noise localized at time of trial prompt (event) compared to white noise localized at the ends of trials. Solid lines correspond to average model output for the best performing trial of each subject, trial and 10 randomly drawn different noise signals. Translucent fill around solid lines indicates a single standard deviation across noise. Trial lengths considered here are 3.5 s, thus for each sample obscured before trial or beginning of trial corresponds to 6 obscured after or at end of trial for the *Obscure Event* and *Obscure Ends* conditions respectively.

To more distinctly see what sections of a trial have the most impact on performance, we plotted the performance of the SCNN model as a function of how much of the trial is obscured by white noise (Fig. 5). We plotted the performance of two different scenarios: when the obscured portion was centred around the moment the subjects were cued to perform their task, labelled *Obscure Event*, and obscuring the ends of the trial, labelled *Obscure Ends*. Obscuring the trial event immediately reduced the performance of the model and steadily underperformed the *Obscure Ends* case. For approximately 250 ms, obscuring the ends of a trial had no effect on performance, indicating that this portion of the data was likely unnecessary. What is of particular interest besides the consistent difference in performance, was the *steady* decrease in performance both models demonstrated. The lack of any particularly sharp and distinct drop within the first half second made it unlikely that some age correlated event-related field (ERF) was solely responsible for the performance we demonstrated, as most ERFs (or event-related potential in the case of EEG) are typically shorter than a half-second^14^. If this was the main contributor to performance, once the entire ERF was fully obscured the performance should have immediately dropped, instead there was a fairly steady drop for the first two seconds. It is of course still possible that an ERF plays a role in classification decisions, but it appeared unlikely that they are a major contributor.

Obscuring the event gave a fairly consistent decrease in performance for at least the first two seconds of obscured points. Least-squares linear regression of this segment found a slope of −0.147 ($p\ll {10}^{-10}$ and correlation *r* = −0.986). Likewise when obscuring the ends, there appeared to be a similar trend in performance between approximately 1 second and 3 seconds. For this segment the slope using least-squares linear regression was −0.173 ($p\ll {10}^{-10}$ and correlation *r* = −0.987). Testing for similarity between the two slopes found that the slope of these lines were not in fact the same ($p\ll {10}^{-10}$, *t*_1394,*two*–*sided*_ = −17.97), so the decrease in performance did not happen identically in these sections.

### Secondary dataset classification

To benchmark how accurate the SCNN and its attention augmented counterpart were compared to other neuro-physiological data, we considered their predictive cability with a standard BCI dataset. The results in Table 5 show that the presented models achieved results that are comparable to state-of-the-art end-to-end machine learning approaches. At first glance there were some increases in classification accuracy, although the augmented SCNN model performed relatively poorly with the augmented dataset. Performing paired Wilcoxon signed-rank tests of both SCNN and Ra-SCNN models with respect to the un-augmented CNN as FBCSP implementation, showed no significant improvement in performance, although the SCNN model with cropping augmentation did show a significant decrease in performance with respect to the benchmark with *V*_8,*one*–*sided*_ = 45 and *p* < 0.002. It appeared that these models were at least as good as a comparable state-of-the-art implementation in a completely unrelated task, excluding the SCNN with cropping augmentations.

**Table 5:** Classification accuracy of SCNN and Ra-SCNN models proposed in this work as compared to the convolutional neural network implementation of a filter bank common spatial pattern (FBCSP) classifier introduced by Schirrmeister *et al*.^25^.

Dataset	Model	Mean %	Dev. %
BCI IV 2a.	FBCSP-CNN	70.9	12.6
	SCNN	72.6	10.7
	Ra-SCNN	73.3	13.6
BCI IV 2a. (+cropping aug.)	FBCSP-CNN	65.6	12.2
	SCNN	35.1	14.2
	Ra-SCNN	66.6	17.1

## Discussion

This work proposes two novel deep neural network architectures that can distinguish between children at different levels of speech development with at least equivalent if not greater accuracy than a feature based-pipeline. We provide evidence that suggest that it is unlikely that classification decisions are made as a result of activity unrelated to the tasks themselves. It is also unlikely that decisions are solely made based on some age-correlated ERFs. The advantage of using these networks is their consolidated training process (they are entirely trained through gradient-descent based optimization without intermediate steps) and as greater steps towards explainable neural networks are made, their parameters may have direct and interpretable connections to the task they are employed against. They also perform quite comparably against the state-of-the-art in a completely separate BCI classification task, making them a general-purpose approach to many encephalographic data.

The finer-grained prediction results show promise that more specific contrasts can be made using our approach and future work will consider how best to deal with continuous phenomena like this. In this work, we have limited our detailed analyses to the binary prediction for the purposes of visualization. Future work will strive to leverage new mechanisms to determine *why* a model decides to classify a trial as a certain category rather than another.

Towards determining if the /*pah*/ and /*pah tah kah*/ vocalization trials or the verb generation experiment trials had a greater impact on performance in the end-to-end learning, we trained the raw SCNN model using these datasets separately. Interestingly they seemed to have difficulty outperforming random chance on their own (fine-grained classification in earlier model exploration). This suggests that despite being slightly different experiments perhaps at the very least the increase in training data is crucial to enabling the performance of these more powerful ML models.

We also spent some time exploring CNN architectures that developed features using data across time and space, but found very little success with these methods, (although these experiments were preliminary, such that their parameters were selected heuristically). Interestingly the failures were generally not the result of over-fitting, as they found fitting to the training data surprisingly difficult (they could however memorize smaller subsets of the data). On the other hand, interpolating the channel data into a series of images rather than a series of channel vectors was *particularly* susceptible to overfitting. Unless considering datasets with many more training points, or employing some more effective regularization techniques we would not recommend proceeding with spatially projected data in future work.

We train the early convolution layers in conjunction with the subsequent LSTM+attention layers for our attention augmented model. Previous work favours using pre-trained networks, but this is mostly due to the ubiquity of pre-trained image networks which make the need for re-training an image network unnecessary and time-consuming. This amounts to an added challenge of training a subset of the network to calculate a range of spatial and temporal filters, and then learn how to weight the sequence of these features. Future work would likely benefit from separating these tasks more to follow previous successes with attention mechanisms. For example the formulation of attention that we borrow from most^15^, is a transduction task that focuses on different aspects of a *pre*-*trained* CNN for image classification and then answers questions about the images using a LSTM+attention layer. This allows them to keep a relatively small training set (one that is less than one million examples) despite such a complex task. An interesting direction for future work might be to develop some general MEG/EEG applicable early layers out of the combination of many different open-access datasets, and then employ LSTMs with attention mechanisms for fine-tuning for a specific task.

Although no statistically significant conclusions can be made as to the power of the SCNN model versus its attention augmented counterpart, we should point out that when employing the cropping augmentation, particularly with the secondary dataset, and to a lesser extent primary task, the SCNN performs poorly as compared to the Ra-SCNN. This makes some intuitive sense, since the attention mechanism should allow for some insensitivity with respect to event offset, in a more powerful sense than the SCNN, which would rely on a later pooling layer for example to provide this functionality (and thus have a limited range). In practice however, the SCNN trained with cropping augmentation performed much better in the seven-way classification task than any other model.

As we mention below in our description of maximal activations, previous successes with this technique, such as in Yosinski *et al*., use a heuristic combination of regularization methods. These penalties act as priors so that the generated data fits the form of real data^13^. An interesting example Yosinski *et al*. provide, is the use of Gaussian blur penalization that penalizes the production of images with high spatial frequency^13^. We notice that in fact in our work, the spectrograms we calculate in fact do demonstrate some strangely distributed high frequency activity. It may be practical to not just begin with data that have 1/*f* spectral density as we do with our data, but to also penalize deviations from this expectation. There may be more that can be done to regularize these generated data and this warrants further study.

Future work that investigates maximal activations should also take steps to gauge event related synchrony/desynchrony in the context of generated data, as the current consideration of spectrograms alone is fairly limited. Work in this direction may prove particularly interesting since convolution operations as implemented by most machine-learning libraries (including, with respect to our implementation, Tensorflow https://www.tensorflow.org/) in CNNs are in practice calculating a correlation measure^16^.

These results are interesting as a demonstration of the ability of end-to-end models to predict speech development, but neural networks show perhaps even greater promise as computational modelling techniques that to some degree mimic the human brain. For example^17^ compare the activations of an image classifier CNN and the MEG and fMRI activity of 15 subjects viewing the same test objects as the CNN. The architectures we present here are very general-purpose, given that they mimic the BCI pipeline, but future work should consider deep neural networks that mimic architectures that follow speech models with suggested neural substrates, such as the DIVA model^18^. These could be used to predict speech output or articulator positions with data such as ours, and explore their connection to the hypothesized brain structures.

## Methods

### Primary dataset: Synchronized MEG and speech recordings

These data were originally recorded to examine age- and sex-related developmental language differences in children by Doesburg *et al*.^19^ and Yu *et al*.^5^. Table 6 summarizes participant demographics. Each participant spoke English as their first language and had no known or suspected histories of speech, language, hearing, or developmental disorders, according to their parents. The acquisition protocol was approved by the SickKids Research Ethics Board (REB #1000016645) which acts in accordance with the guidelines established by the Tri-Council Policy on Ethical Conduct for Research Involving Humans. All participants or their parents gave written informed consent. Children unable to read the consent form provided informed verbal assent. Prior to the experiment, children received two standardized language clinical tests: the Peabody Picture Vocabulary Test (PPVT)^20^ and the Expressive Vocabulary Test (EVT)^21^. All children’s scores were at or above expected scores for their ages on the PPVT and EVT, and their speech showed neither signs of articulatory difficulties nor any significant effect of age. In total, 80 participants were right-handed, 5 were left-handed, and 7 were ambidextrous, according to the Edinburgh assessment^22^, there was no significant variation of handedness with age.

**Table 6:** Participant demographics, across stimuli types.

Stimuli	Mean Age	Age range	Subjects	Trials	M/F Split
/*pah*/	10.73	4.1–18.1	89	115	0.45/0.55
/*pah tah kah*/	11.01	4.1–18.1	83	115	0.45/0.55
VG	13.23	5.7–18.0	28	81	0.42/0.58

The two speech tasks involve considerable participant overlap.

Three distinct speech-elicitation stimuli were used. The first two were of the monosyllable /*pah*/ and the multisyllabic sequence /*pah tah kah*/, respectively. These were simple enough for young children and are part of the diadochokinetic rate (DDK) test, which has been used to evaluate neuromuscular control in motor speech disorders. Prior to acquisition, the experimenter demonstrated the productions of each stimuli, without word-like prosodic patterns. A small white circle was displayed on the monitor as a cue to begin performing the speech-elicitation. The circle was present on the monitor for 200 ms. The third experiment was an overt verb generation (VG) task in English, where subjects were presented with an image with which they were familiar, and were asked to produce a verb associated with the object^19^. Image stimuli were presented to subjects for 500 ms, and also served as cue to begin the task. Between stimuli, participants were instructed to focus on a small white cross to minimize eye movements.

Recordings were made with each participant lying supine in a magnetically shielded room in the Neuromagnetic Lab of the Hospital for Sick Children in Toronto, using a CTF whole-head MEG system (MEG International Services Ltd., Coquitlam, BC, Canada). To minimize movement with younger subjects, their heads were padded to restrict space, and they were given very clear instructions to stay still. If there was excess movement, the acquisition of trials was restarted, and if the subject continued to move the participant would have been removed. We considered only subjects and recordings that passed these requirements. The system recorded 151 MEG channels, and a single audio channel, with a sampling rate of 4 kHz. We performed a bare-minimum cleanup of the data where we resampled MEG signals at 200 Hz, and band-pass filtered between 0.5 Hz and 100 Hz, to remove offsets and accommodate the canonical ranges of *δ*, *θ*, *α*, *β*, and *γ* activity. Electrooculography (EOG) artifacts were removed using second order blind source identification as provided by EEGlab^23^. Each trial consisted of 1.5 seconds before to 2 seconds after the onset of event cue, for trials of 3.5 seconds in length, with the exception of the training augmentation through cropping explained below.

Using these data, Doesburg *et al*.^19^ predicted language ability as an increase in network synchrony, particularly in the theta band with the increase of age during verb-generation (VG) tasks in children and adolescents. They observed a significant increase in the number of synchronous regions with older adolescents, compared with younger children. Yu *et al*.^5^ specifically focused on four regions of interest: inferior frontal regions, dorsolateral prefrontal regions, temporal-parietal regions, and superior temporal regions. They noticed distinct profiles of localized de-synchrony over time in VG tasks for children within five age ranges (i.e., 4–6, 7–9, 10–12, 13–15, and 16–18 years of age).

### Supplementary dataset: BCI Competition IV, Dataset 2a

In an effort to compare the potential success of our end-to-end models to previous work, we also trained some of our proposed models using the BCI Competition IV EEG Dataset^24^. This dataset has been featured in several other attempts to apply neural networks trained end-to-end to neurophysiological data^25–28^ and is freely available online: http://bnci-horizon-2020.eu/database/data-sets.

These data consist of EEG recordings of 9 subjects performing 4 different imagined motor tasks: left hand, right hand, both feet, and tongue. These recordings were broken up into two separate sessions for each subject recorded on different days. Each of these sessions consisted of 6 sets of 48 trials separated by a short break, where the 48 trials were 12 executions of each of the 4 tasks. Thus a total of 288 trials were recorded per session. One session is considered training data, and the second is used as evaluation requiring some carry-over performance between days. The recordings consist of 22 EEG electrodes, and 3 monopolar EOG electrodes, all recorded at 250 Hz and bandpass filtered between 0.5 and 100 Hz. Additionally, a 50 Hz notch-filter was used to minimize line noise. The trials themselves are available as 6 second recordings, where the first two seconds consist of presenting each subject a fixation point. At 2 s through 3.25 s, a task stimuli was presented to the subjects. There is additionally some EOG-only trials per session that we discarded.

### Training Augmentation Through Cropping

To augment the number of training points available, the entire trial was split into multiple training examples by taking subsections of each trial that still included the event onset. Effectively, rather than one training datapoint for each trial, a sliding window smaller than the length of the trial was used to crop many points, where each point had the event onset localized in a different place. The premise behind this augmentation was that with the event localized in different places, an architecture like a convolutional or recurrent network could learn temporal filters that were agnostic of a specific onset time and thus should be more generalizable. Previous work that has taken a similar approach include Schirrmeister *et al*.^25^ and Sun *et al*.^28^, but alternatively to our work, they assumed a convolutional input stage and provide variable temporal length training points rather than fixed length crops. We elected to use a sliding window of 2 seconds, which resulted in (3.5 *s* − 2 *s*) × *SamplingFrequency* possible crop location per training point, or a possible augmentation of 300:1.

### Detailed neural network architectures

#### Spatial summary convolutional neural network (SCNN)

The critical innovation of the SCNN architecture is to enable multiple layers of non-linear spatial convolutions and temporal convolutions to be performed separately. This results in a set of spatial filtering components that can be examined independently from temporal features. This also mimics the feature-based approach pipeline of: (i) spatial (channel) mixing, (ii) applying filter banks to mixed components to develop a set of features, and then (iii) finally classifying with these features. In this architecture, the spatial convolutions ultimately span the length of the incoming channels, but rather than a single linear layer, we introduced non-linearity and multiple convolution layers that work together to construct a more flexible spatial filter, but one that still employs weight sharing at each step. We selected a hyperparameter indicating the depth of the spatial filter, and then stacked convolutional layers, where each layer reduces the spatial dimension until the final layer completely collapses it. A number of temporal filters (i.e., convolutions over the temporal dimension) were then applied to the new spatial mappings (the number was also a selected hyperparameter), and an optional average pooling layer was applied to reduce the number of parameters and effectively low-pass filter these new features. Finally, the resulting features were flattened into a single vector and a fully connected neural network (FFNN) classifier was used as an output stage, with the number of layers determined again through hyperparameter search.

**Figure 6:**
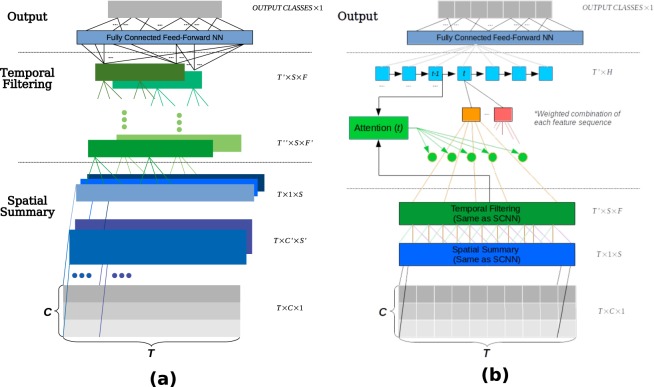
The two general CNN architectures we propose. Processing of trials flows from bottom to top, where the grey arrays represent an incoming trial of *C* channels and *T* samples (where for our primary dataset: *C* = 151 and *T* = 400). (**a**) Spatial summary convolutional network (SCNN), the architecture is broken down into three stages. The *Spatial Summary* stage performs convolutions exclusively across channels at each timepoint, thus not impacting the number of samples (the second dimension *T* remains unchanged). Likewise, the *Temporal Filtering* stage performs convolutions exclusively across the time dimension. The output stage flattens the resulting output of the previous stages and uses a feed-forward network for final classification. The colours are used to distinguish between sequences produced as a result of different learned convolution kernels at each stage. (**b**) The Ra-SCNN network, which extends the SCNN presented in (**a**). The output of the *Temporal Filtering* stage in (**a**) is fed as input to an LSTM enhanced with attention weighted input layer. Finally the output of the LSTM uses a feed-forward network for final classification.

The first few layers of the CNN can be seen as a mapping from *R*^*T*×*C*×1^ to *R*^*T*×1×*S*^ (the spatial filtering) to *R*^*T*′×1×*F*^ (the temporal filtering) where *T*′ ≤ *T*, *C* is the number of recording channels, *S* is the number of spatial transformations, and *F* is the number of temporal filters. In other words, a set of *S* non-linear spatial transformations were applied to the *C* channels, and then a set of *F* temporal filters were applied to these new sequences, as can be seen in the diagram presented in Fig. 6(a).

Since the actual depth (of spatial filtering and temporal filtering operations) changes depending on the configuration selected by the hyperparameter search, consider an example architecture with two layers of spatial steps with *K*_1_, *K*_2_ kernels for each layer respectively. Note that there are no bias terms included as batch normalization^29^ renders them redundant and is employed throughout. Let *X* be our input data with dimensions *T* × *C*, where *T* is the length of our temporal sequence and *C* is the number of channels recorded. Also, as a result of the two spatial layers, we intend to produce the output *X*_*Spatial*_ with dimensions *T* × *K*_2_ where *K*_2_ is the target number of *spatial components*. In the first layer, and *j*^*th*^ kernel ${w}_{{L}_{1}}^{j}$, has length *C*′, where *C*′ < *C*, and the output of filter *j* of the first layer at time *t* is then:

1 $${X^{\prime} }_{t,i,j}=f(\sum _{k=0}^{C^{\prime} }\,{w}_{{L}_{1k}}^{j}{X}_{t,i+k})$$

where *X*′ is a three dimensional tensor of shape *T* × (*C* − *C*′) × *K*_1_ and *f*(*x*) is the neuron’s non-linear function of choice. After the hyperparameter search detailed later, the SCNN model uses a scaled exponential linear function (SELU)^30^. There is no integration of any temporal information besides *t*, and that for each kernel there are *C* − *C*′ sequences of length *T*, of which only the *i*^*th*^ is shown above. Next, the second layer consists of kernel vectors ${w}_{{L}_{2}}^{s}$ of length *C* − *C*′, which determine component *s* of *K*_2_ at time *t* as follows:

2 $${X}_{Spatia{l}_{t,s}}=f(\sum _{i=0}^{C-C^{\prime} }\,{w}_{{L}_{2i}}^{s}{X^{\prime} }_{t,i,j})$$

The temporal filtering operations behave like eq. 1, but each kernel in the temporal layer is applied across the *temporal* dimension of each kernel activation sequence of the previous layer.

#### SCNN model augmented with attention focused recurrence (Ra-SCNN)

The Ra-SCNN is an extension of the SCNN architecture, that uses an LSTM and attention mechanism after the spatial and temporal steps to provide the network with a stronger ability to focus on aspects of the signal. In our experiments, we employed an architecture (diagram shown in Fig. 6(b)) that is very similar to the encoding stage employed in Zhu *et al*. for the purpose of image-directed question answering^15^. Those authors used a pre-trained CNN and an LSTM-based encoder that was fed attention weighted inputs. Their attention mechanism provided an average weighting of the different convolutional feature maps which are combined with one-hot encoded word vectors representing the words in the questions.

In contrast, our implementation does not use a pre-trained CNN, but trains convolutional layers at the same time as the rest of the model. The attention serves as a mechanism to closely consider parts of the sequence depending on the state of the LSTM. Where for some feature *j* and point *t* in a sequence of *T*, a new value for *j* is calculated as the weighted sum of entire sequence of that feature.

3 $${e}_{t,j}=a({f}_{0,j},\ldots ,{f}_{T-1,j},{h}_{t-1}),$$

where $a()$ is an arbitrary function (we use a multi-layer network similar to the original formulation in^15^, but the number of layers used is a hyperparameter determined during search, and units per layer equal to the number of hidden units with the associated LSTM.). These vectors are normalized (so that they sum to 1, and range between 0 and 1) using the softmax function. This produces the *soft*-*attention* weights *α*_*t*,*j*,*t*′_ for each feature *j* at time *t*′:

4 $${\alpha }_{t,j,t^{\prime} }=\frac{\exp ({e}_{t,j,t^{\prime} })}{{\sum }_{i=0}^{T-1}\,\exp ({e}_{t,j,i})},$$

Finally the new attention-weighted feature $\hat{f}$ is:

5 $${\hat{f}}_{t,j}=\sum _{i=0}^{T}\,{f}_{i,j}{\alpha }_{t,j,i}$$

So the intent is that the LSTM develops some sort of sequential processing that does not need to progress temporally sample by sample, but is flexible to consider any combination of samples that may become appropriate. In our case specifically, the attention mechanism pre-processes the output of the spatial summary and optionally (a hyperparameter) filtering over time.

6 $${x}_{t,i}^{In}=\sum _{t=0}^{T^{\prime} }\,{\alpha }_{t,i}{X}_{t,i}^{SCNN}$$

We selected whether to use the full LSTM output sequence, or exclusively the last state output as part of our hyperparameter search, and optionally have several fully connected layers before the prediction layer.

### Obscuring Profiles

To produce Fig. 5 we averaged *profiles* created for each test subjects in the test dataset, for each of the highest confidence (defined here as largest correct class output) test points within each task the subject performed. Each *profile* consisted of 10 different uniform noise signals that grow from the event out to the ends in the case of *Obscure Event* or from the ends towards the event in *Obscure Ends*. Obscuring consists of a new point created out of the original signal alone for all points that are not obscured, and a weighted combination of the original signal and a noise signal, at a relative weighting of 1:1000 respectively for the section that is obscured. This obscuring technique then *grows* by the addition of a single sample to the set of obscured points (and the respective removal of that point from the un-obscured set). For each sample obscured and each of the 10 noise signals, these modified points are fed to the pre-trained model and plotted. Since the event prompt is localized at *t* = 0.5 *s* within a trial that is 3.5 seconds long, the growing process proceeds at a rate of 6 samples to 1. Where 6 samples are added after the event at *t* = 0.5 *s* for each added before in the case of *Obscure Event*, and 6 samples are added to the end of the trial for every 1 added to the beginning in the case of *Obscure Ends*.

### Model analysis and visualizations

Explaining *what* a trained neural network has learned is an ongoing area of research, and a particularly important one. For neural-network based models to be significantly useful beyond their successes as classifier tools, understanding what they are doing is crucial. We took an approach that generates synthetic data by maximizing the outputs of a trained model with respect to these synthetic data (with no reliance on training points in particular), similar to that presented by Yosinski *et al*.^13^. We selected this approach to focus on what type of input is preferred by key points in our models, and involves performing regularized gradient-ascent of an output *f*_*o*_(*x*) within the fully trained model with respect to the desired input *x*. So that we generate a synthetic datapoint or activation *x*_*gen*_ where:

7 $${x}_{gen}=\mathop{{\rm{\arg }}\,{\rm{\max }}}\limits_{x}({f}_{o}(x)-R(x))$$

Here we summarize any regularization penalties as *R*(*x*). Yosinski *et al*. show that crucial to the success of this technique is to ensure good prior distributions on the synthetic data^13^, so in this spirit we begin with randomly initialized data that have a spectral density that decreases with 1/*f* and zero mean, thus conforming to general encephalographic signals. We also use L2 regularization on our data-points which helps prevent unbounded growth during the ascent which in effect prevents some strongly relevant features from eclipsing some features that are important but less impactful on the outputs. The iterative maximized input value is then:

8 $${\hat{x}}_{gen}\leftarrow \eta ({\hat{x}}_{gen}+\frac{\partial }{\partial x}({f}_{o}(x)-\theta \cdot \sum _{i}\,{x}_{i}^{2}))$$

where we heuristically select a step increase of $\eta =0.2$ and L2 regularization of *θ* = 0.05. We then iterate up to 10,000 times, stopping if no progress is made for more than 5 steps. Additionally we normalize the derivative in eq. 8 for each step by its RMS value to make more stable (less oscillatory) progressions.

We generated activations for two sets of input-output point pairs of the trained (using the binary classification task and primary dataset) end-to-end models. The first was between the model’s overall input, and the end of the final-most spatial convolution layers, these were then interpolated (using bilinear interpolation) across the true channel locations of our MEG machine to demonstrate a rough localization of spatial components/mixing. The second input-output pair was between the input to the first temporal convolution (the output of the last spatial) and the final model output (classification stage). The goal of the second pair was to generate data that emphasized desired temporal features. This was then illustrated by calculating power spectral density spectrograms (Hann windows with 50 samples overlap and 64 FFT bins) between the output of the spatial stage and the class prediction layer.

### Comparison features

For comparison, we perform a feature-engineering pipeline for use as a comparison against our proposed models. We first apply info-max independent component analysis (ICA)^31^ to determine statistically independent sub-components of the MEG recordings, across all subjects. These new components and a parallel recorded audio channel are then separated into windowed epochs corresponding to frames −500 ms to +1500 ms around the onset of the stimuli prompt. We extract 156 acoustic features and 4681 MEG features from each epoch using openSMILE^32^. These are calculated using 50 ms rectangular windows, with a 25 ms overlap resulting in 79 windows per datapoint. Features to represent spectral activity are calculated for each window, using a fast Fourier transform (128 points for the 4 kH*z* audio recordings, and 8 points for the 200 H*z* MEG channels)) and linear predictive coding coefficients. Additionally, the summary statistics: mean (also absolute mean, quadratic mean, and aforementioned means calculated using only non-zero values), variance, skewness, and kurtosis are calculated. Finally, the root-mean-squared and log of the signal energy are also calculated for each window. Unique to the MEG channels we also calculate an autocorrelation function (ACF) calculated using the fast Fourier transform (FFT) and its inverse (iFFT) for window *w*:

9 $$ACF(w)=iFFT(|FFT(w){|}^{2})$$

As a result of the number of features extracted for each MEG trial, the number of features greatly outnumbers the number of training points available. As this reduces the efficacy of convex optimizations, we first select a sub-set of features to use during the training process. We use standard Pearson correlation between the MEG features and age, and highlight 172 features with *p*-values at *α* = 10^−5^ after Bonferroni correction, and correlation coefficients abs(*r*) > 0.2. These 172 features include the autocorrelation features of eight ICA components (including the first (highest entropy) component) in addition to the entire available frequency spectrum of four of these components.

### Model training procedures

Each model is tested using 5-fold cross-validation against a held-out group of 9 subjects, only one of which did not perform the verb generation task. The test subjects are selected to have a similar distribution of the number of trials in each age range. The remaining subjects are ordered based on the number of trials they performed and their trials are distributed across the 5 folds in this order to create an approximately equal number of trials in each fold. The range in number of training points between folds was 3838 through 4078. We quantitatively evaluate the similarity between the 5 folds and test set using a K-sample Anderson-Darling test, where we found *A*^2^ = −0.271 with *p* = 0.61. In the supplemental dataset, the training versus test datasets are separated in advance for intra-subject training. Due to the imbalance of points between verb-generation and the other two tasks, a loss penalty was applied to each output class equal to $1-\tfrac{{n}_{class}}{{n}_{total}}$ during all training stages of the task prediction experiments.

We use Keras (https://keras.io/) with a Tensorflow (https://www.tensorflow.org/) backend to build all of the discussed models, and use either stochastic gradient descent or the Adam optimizer^33^ when performing back-propagation updates. The fine-grained prediction of seven age targets, target employed *label*-*smoothing*^34^ where a target distribution is used rather than *one*-*hot* encoded labels for the seven classes. In practice this meant setting the true label to 0.6 and its neighbouring classes to 0.2 (and at the upper and lower ends we set the true label to 0.8 with its neighbour again 0.2). We select between three different activation functions during hyperparameter search: the rectified linear unit (ReLU)^35^, the exponential linear unit (ELU)^36^, and the scaled ELU (SELU)^30^. All layers are batch-normalized^29^ after activation. We additionally employ L2-norm weight regularization for all trainable weights, excluding those of RNNs. We apply dropout^37^ after all fully-connected layers, and both max-pooling and spatial dropout^38^ to all convolutional layers. These techniques are not necessarily applied to the FBCSP-like model from^25^, which is reproduced by examining their source code.

All hyperparameter selection is done using the tree-structured Parzen estimator implemented in Hyperopt^39^ including: learning rate, regularization penalty, dropout rates, number of adjacent activations to pool, number and layers of hidden units, receptive fields, and activation functions for all appropriate models. Hyperparameter searches are performed for 100 iterations.

### Access to Collected Data and Source Code

Code to use the SCNN and Ra-SCNN models is available at https://github.com/SPOClab-ca/SCNN. The models are implemented with Keras (https://keras.io/) with the intent that they can be dropped in as an easy-to-use module by an audience with some familiarity with Python and some relevant dataset. Necessary previous experience with neural networks is minimal. We have also included code to develop maximal activation components, and activation spectrograms for a trained SCNN model. For access to the data, please contact the corresponding author.

## Supplementary information

Supplementary Figure S2
