# Supplementary material for: Machine learning for MEG during speech tasks
Source: Sci Rep. 2019 Feb 7;9:1609. doi: 10.1038/s41598-019-38612-9 (PMC6367450; doi:10.1038/s41598-019-38612-9)
Supplement: Supplementary file 1 — Supplementary Figure S2 [file 41598_2019_38612_MOESM1_ESM.pdf]

Supplementary Figure S2.

Machine learning for MEG during speech tasks

Demetres Kostas[1,2,*], Elizabeth W. Pang[1,3,4], and Frank Rudzicz[1,2,5]

[1]University of Toronto; Toronto, Canada
[2]Vector Institute; Toronto, Canada
[3]Hospital for Sick Children; Toronto, Canada
[4]SickKids Research Institute; Toronto, Canada
[5]Toronto Rehabilitation Institute-UHN; Toronto, Canada
[*]demetres@cs.toronto.edu

These diagrams outline the synthetic data and activations acquired through activation maximization for all 60 spatial components of the scrutinized SCNN network. Each sub-figure (row) below consists of the interpolated relative channel weightings on the left, and in the center and to the right the spectro-temporal characteristics of this same component that maximized the < 10 and $>= 10$ classes respectively.

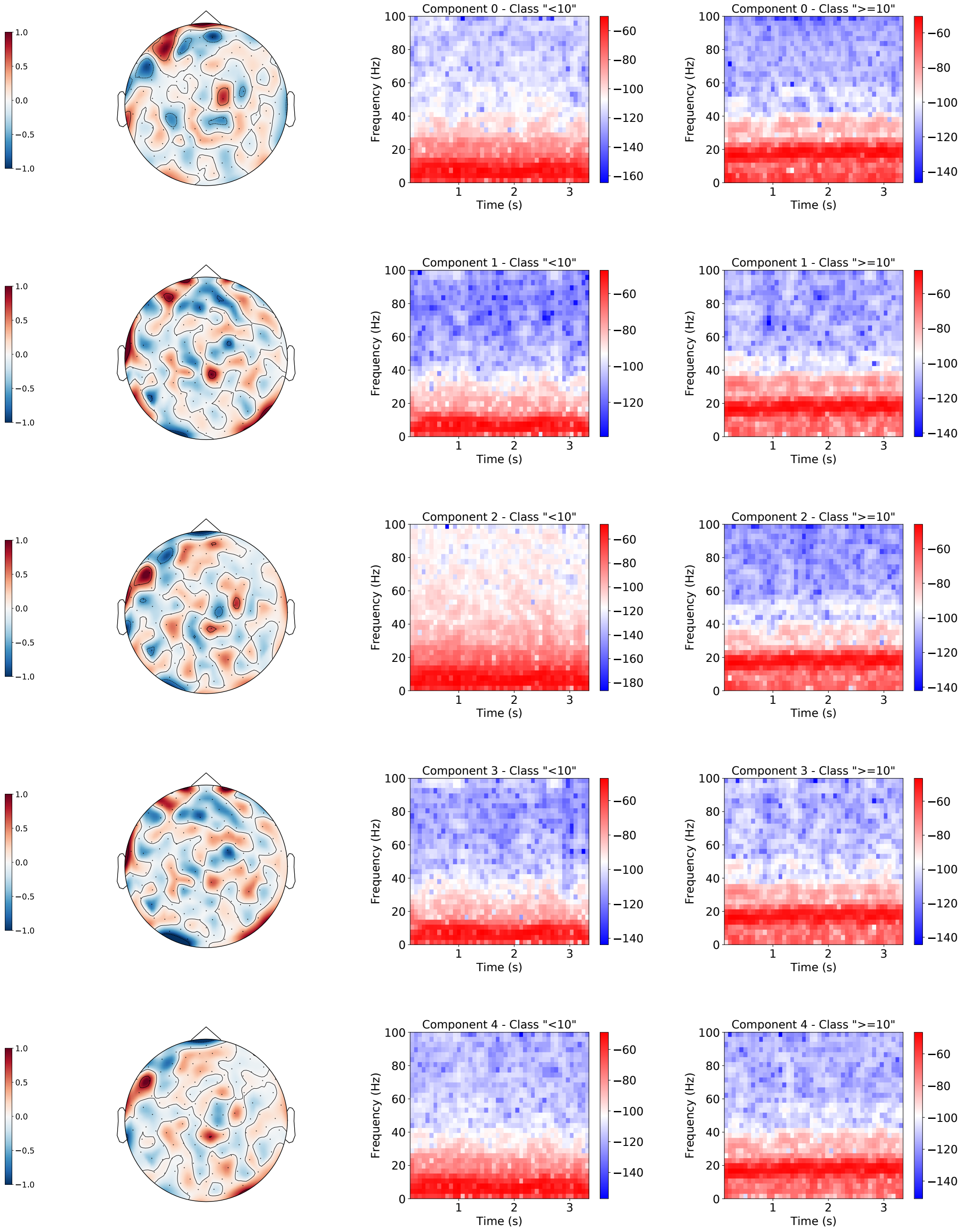

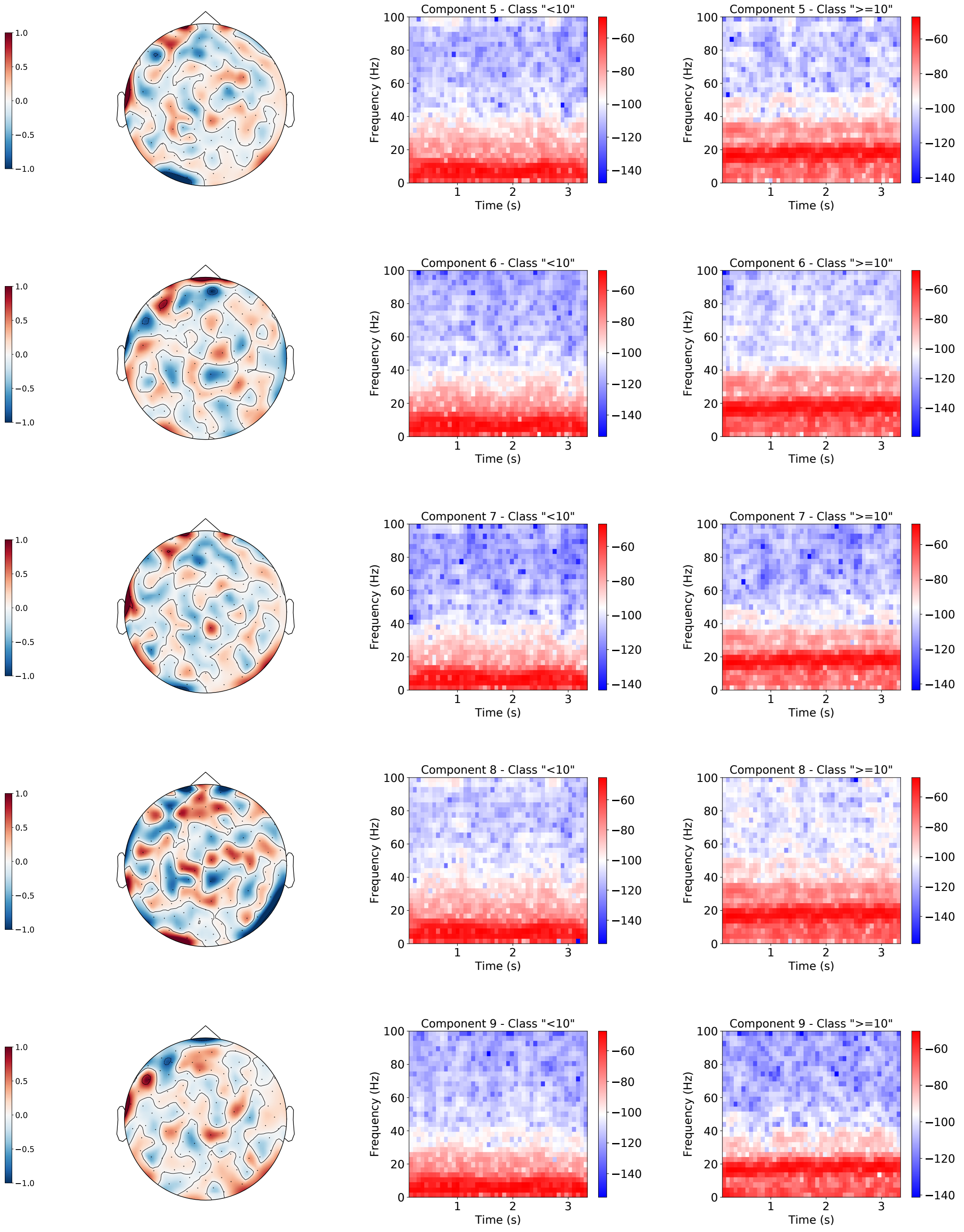
Component 5 - Class "<10"
Component 5 - Class ">=10"
Component 6 - Class "<10"
Component 6 - Class ">=10"
Component 7 - Class "<10"
Component 7 - Class ">=10"
Component 8 - Class "<10"
Component 8 - Class ">=10"
Component 9 - Class "<10"
Component 9 - Class ">=10"
Frequency (Hz)
Time (s)

Figure 3: These diagrams outline the synthetic data and activations acquired through activation maximization for all 60 spatial components of the scrutinized SCNN network. Each sub-figure (row) below consists of the interpolated relative channel weightings on the left, and in the center and to the right the spectro-temporal characteristics of this same component that maximized the < 10 and $>= 10$ classes respectively.

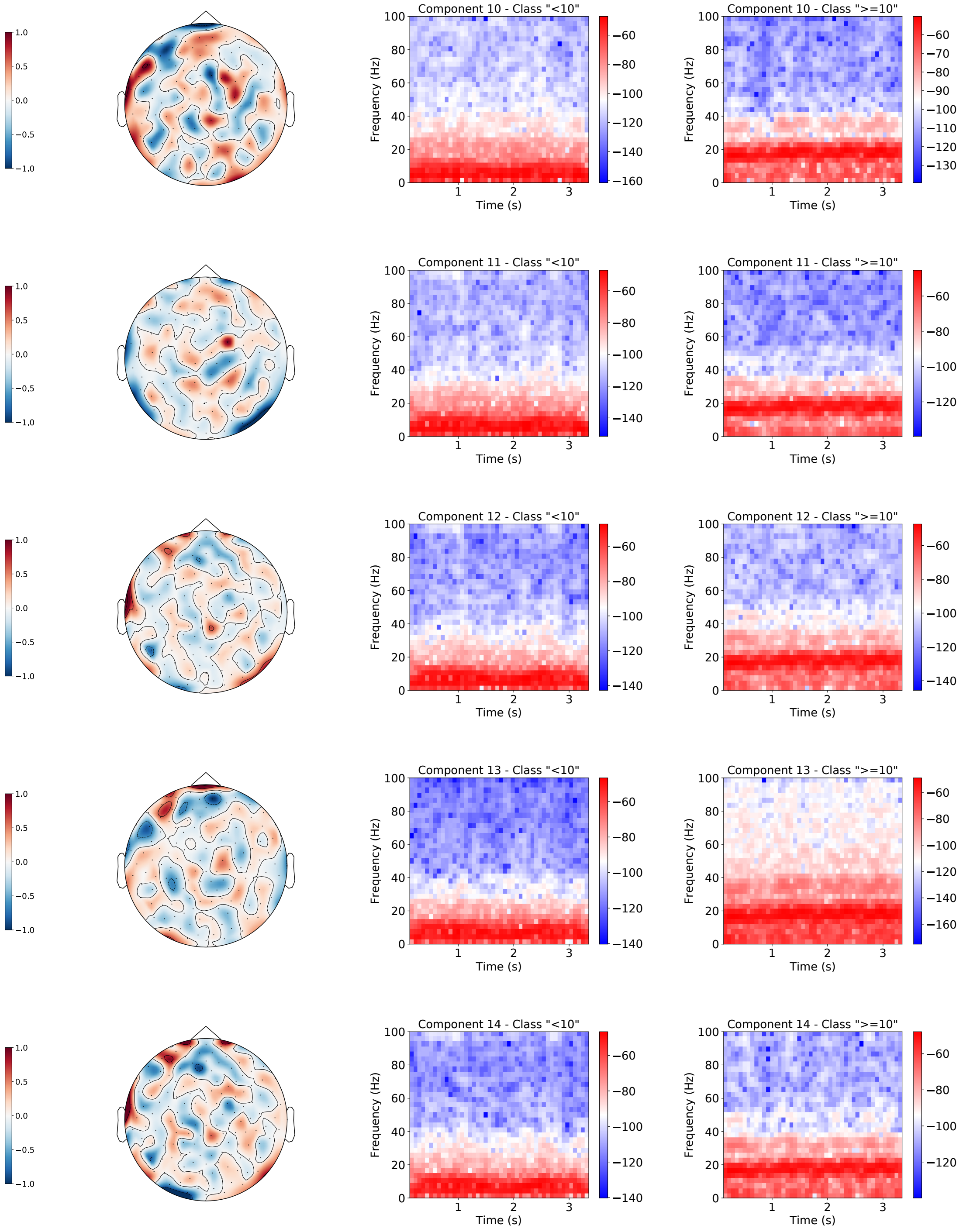

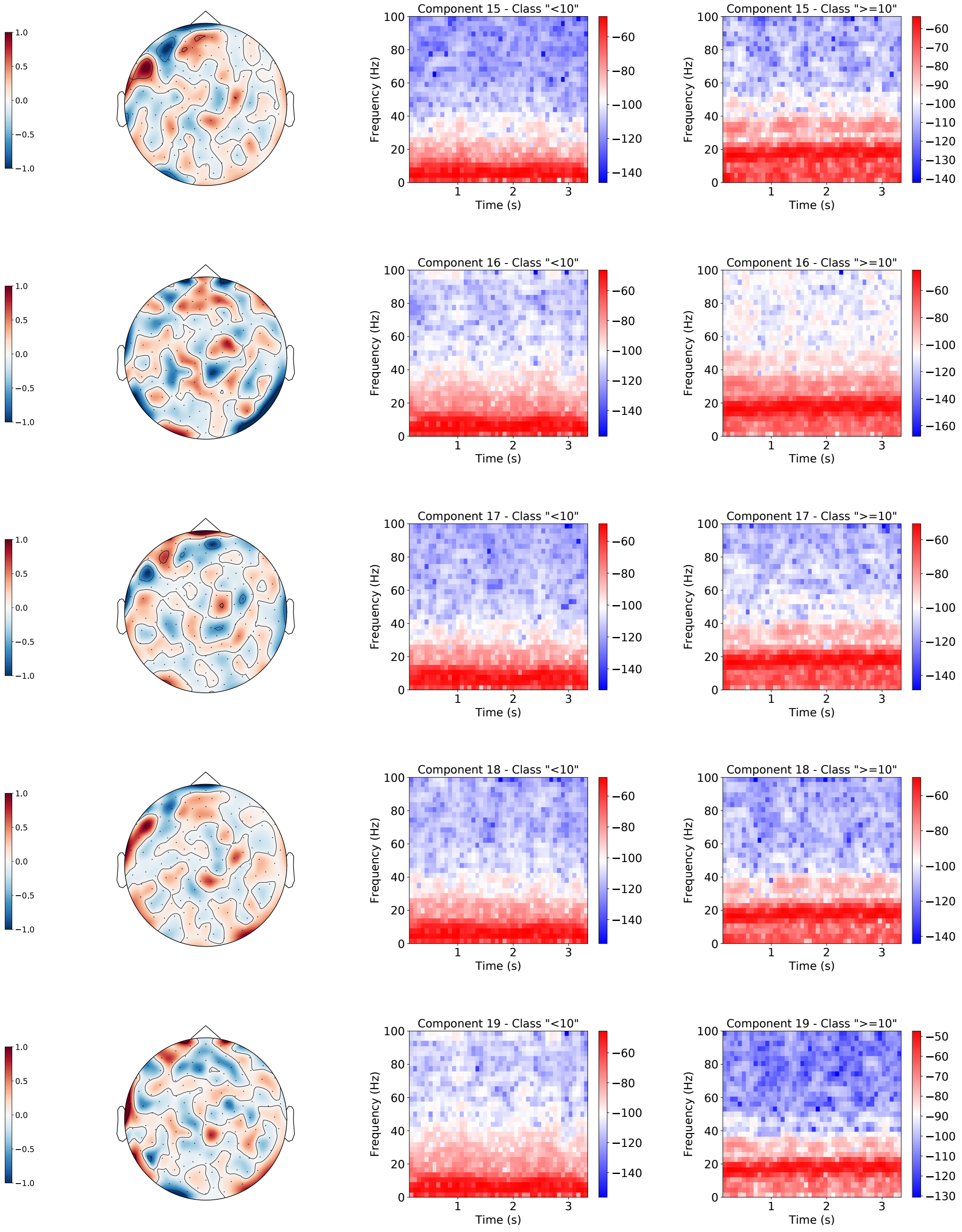
Component 15 - Class "<10"
Component 15 - Class ">=10"
Component 16 - Class "<10"
Component 16 - Class ">=10"
Component 17 - Class "<10"
Component 17 - Class ">=10"
Component 18 - Class "<10"
Component 18 - Class ">=10"
Component 19 - Class "<10"
Component 19 - Class ">=10"
Frequency (Hz)
Time (s)

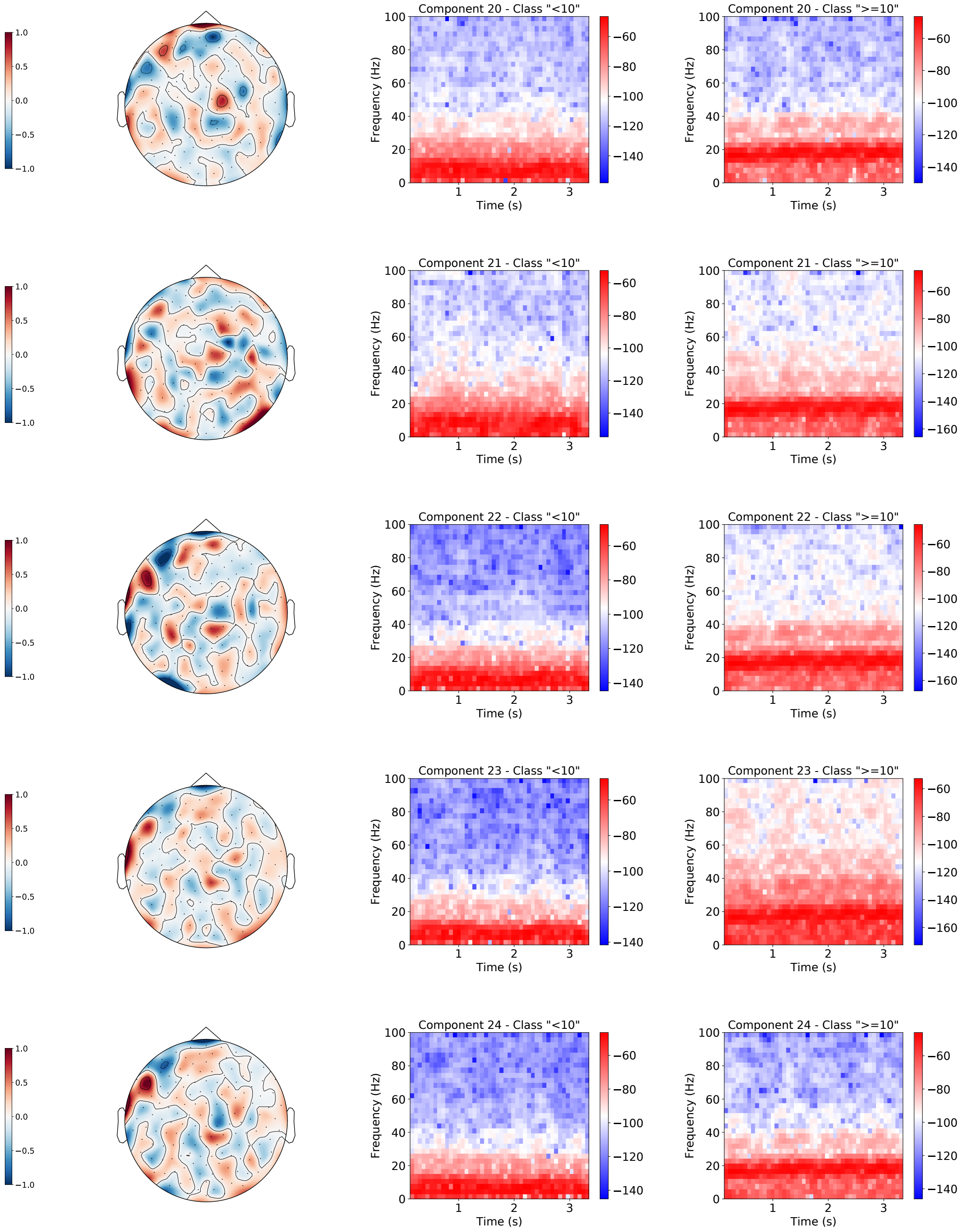
Component 20 - Class "<10"
Component 20 - Class ">=10"
Component 21 - Class "<10"
Component 21 - Class ">=10"
Component 22 - Class "<10"
Component 22 - Class ">=10"
Component 23 - Class "<10"
Component 23 - Class ">=10"
Component 24 - Class "<10"
Component 24 - Class ">=10"
Frequency (Hz)
Time (s)

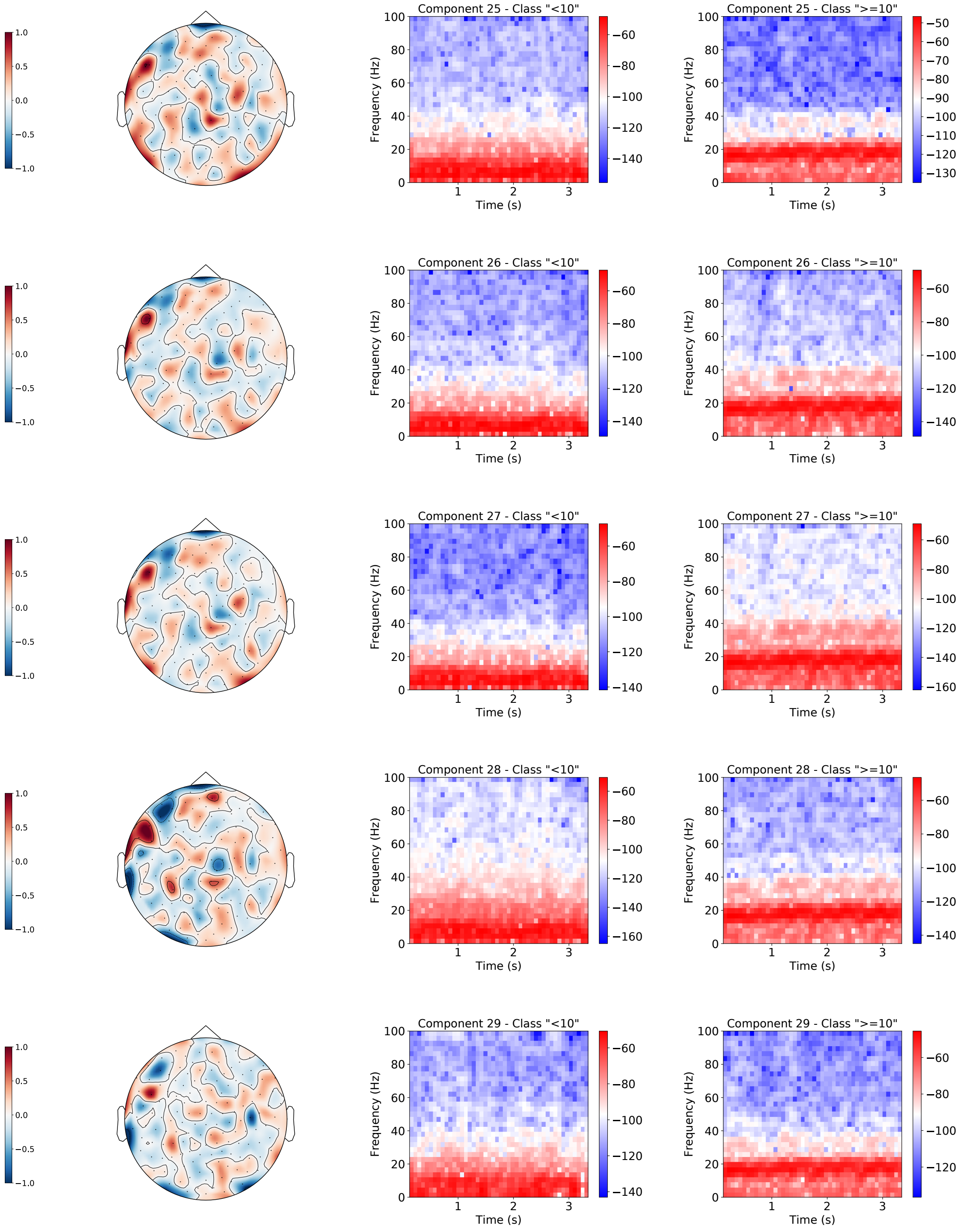

Component 25 - Class "<10"
Component 25 - Class ">=10"
Component 26 - Class "<10"
Component 26 - Class ">=10"
Component 27 - Class "<10"
Component 27 - Class ">=10"
Component 28 - Class "<10"
Component 28 - Class ">=10"
Component 29 - Class "<10"
Component 29 - Class ">=10"
Frequency (Hz)
Time (s)

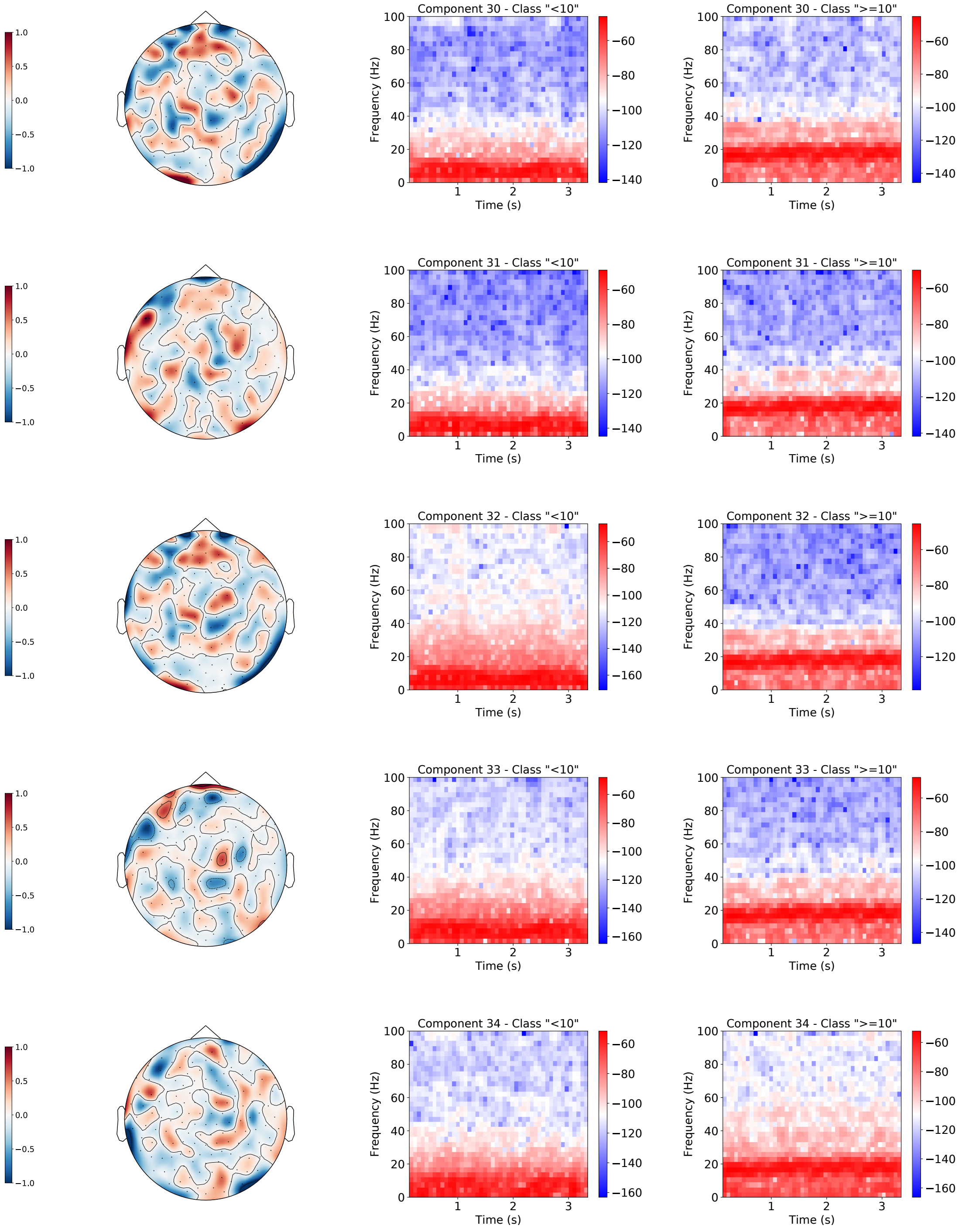
Component 30 - Class "<10"
Component 30 - Class ">=10"
Component 31 - Class "<10"
Component 31 - Class ">=10"
Component 32 - Class "<10"
Component 32 - Class ">=10"
Component 33 - Class "<10"
Component 33 - Class ">=10"
Component 34 - Class "<10"
Component 34 - Class ">=10"
Frequency (Hz)
Time (s)

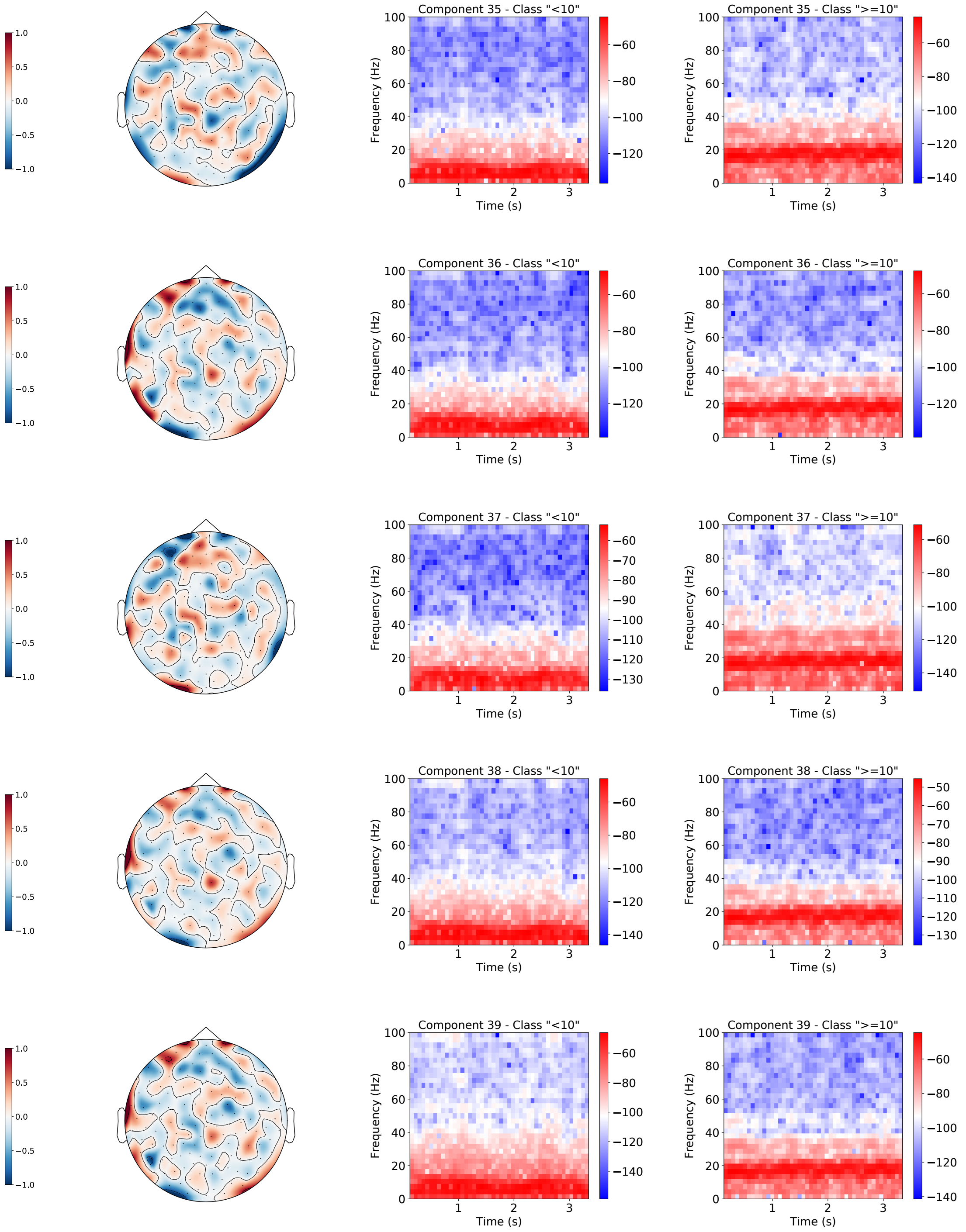
Component 35 - Class "<10"
Component 35 - Class ">=10"
Component 36 - Class "<10"
Component 36 - Class ">=10"
Component 37 - Class "<10"
Component 37 - Class ">=10"
Component 38 - Class "<10"
Component 38 - Class ">=10"
Component 39 - Class "<10"
Component 39 - Class ">=10"
Frequency (Hz)
Time (s)

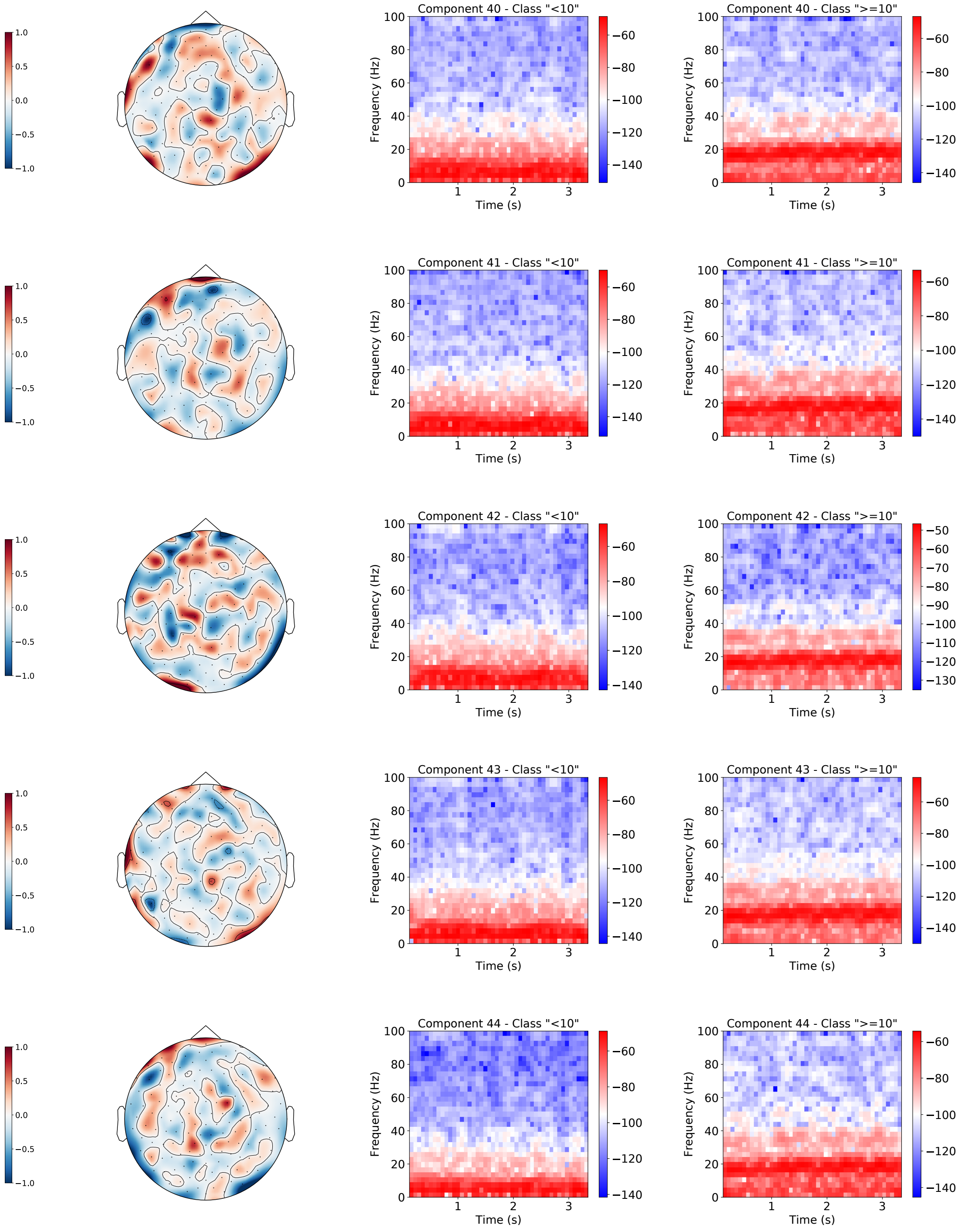
Component 40 - Class "<10"
Component 40 - Class ">=10"
Component 41 - Class "<10"
Component 41 - Class ">=10"
Component 42 - Class "<10"
Component 42 - Class ">=10"
Component 43 - Class "<10"
Component 43 - Class ">=10"
Component 44 - Class "<10"
Component 44 - Class ">=10"
Frequency (Hz)
Time (s)

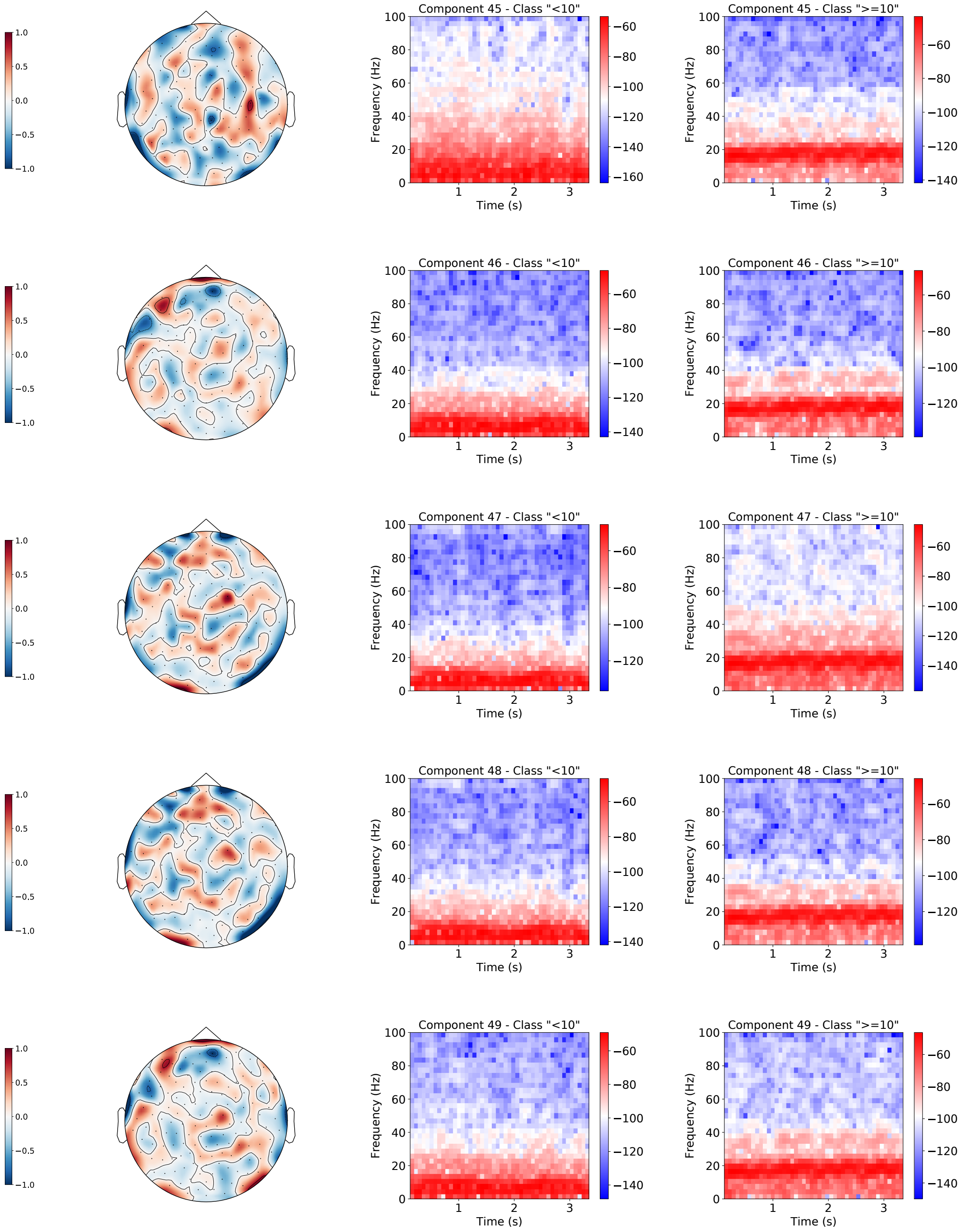
Component 45 - Class "<10"
Component 45 - Class ">=10"
Component 46 - Class "<10"
Component 46 - Class ">=10"
Component 47 - Class "<10"
Component 47 - Class ">=10"
Component 48 - Class "<10"
Component 48 - Class ">=10"
Component 49 - Class "<10"
Component 49 - Class ">=10"
Frequency (Hz)
Time (s)

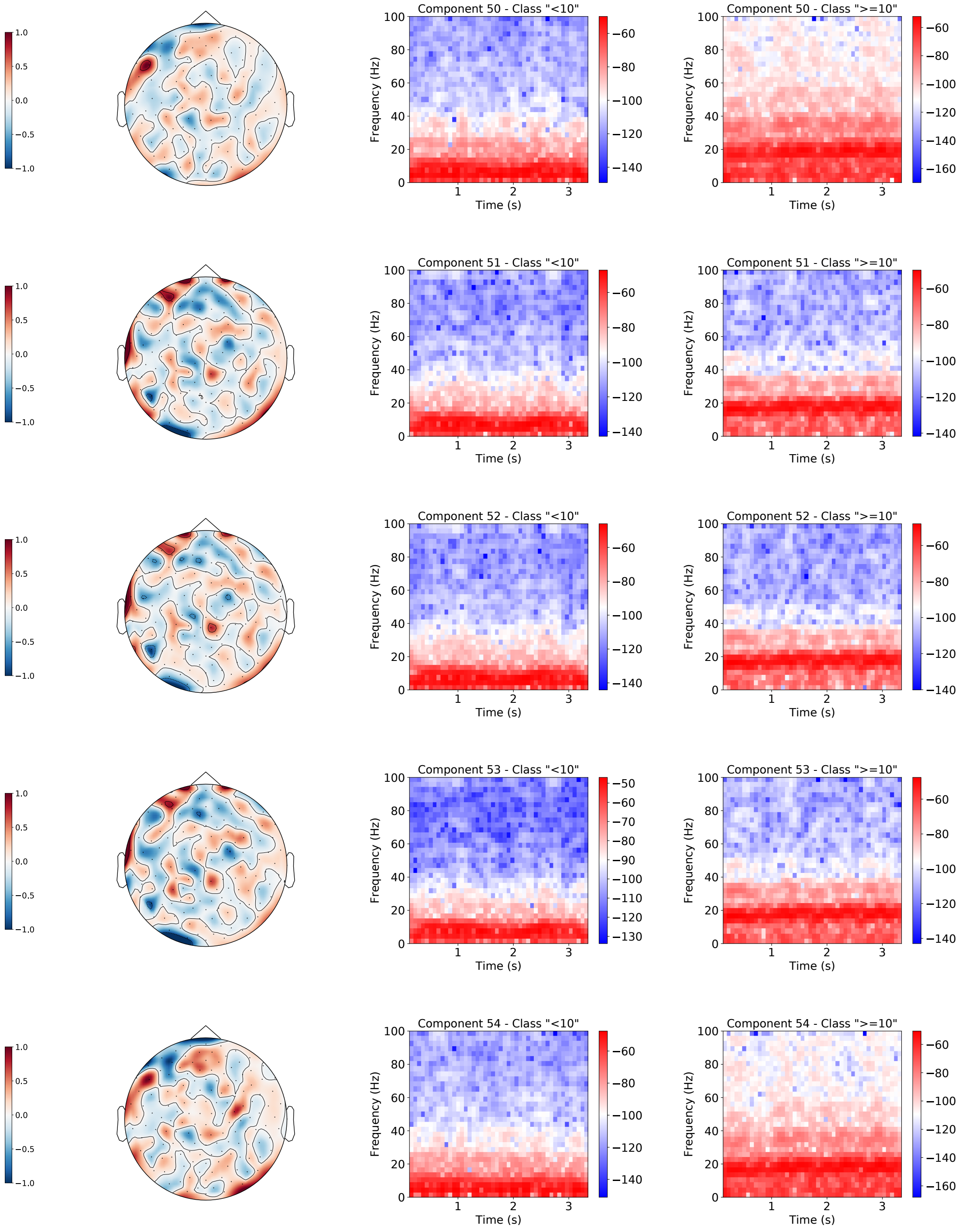
Component 50 - Class "<10"
Component 50 - Class ">=10"
Component 51 - Class "<10"
Component 51 - Class ">=10"
Component 52 - Class "<10"
Component 52 - Class ">=10"
Component 53 - Class "<10"
Component 53 - Class ">=10"
Component 54 - Class "<10"
Component 54 - Class ">=10"
Frequency (Hz)
Time (s)

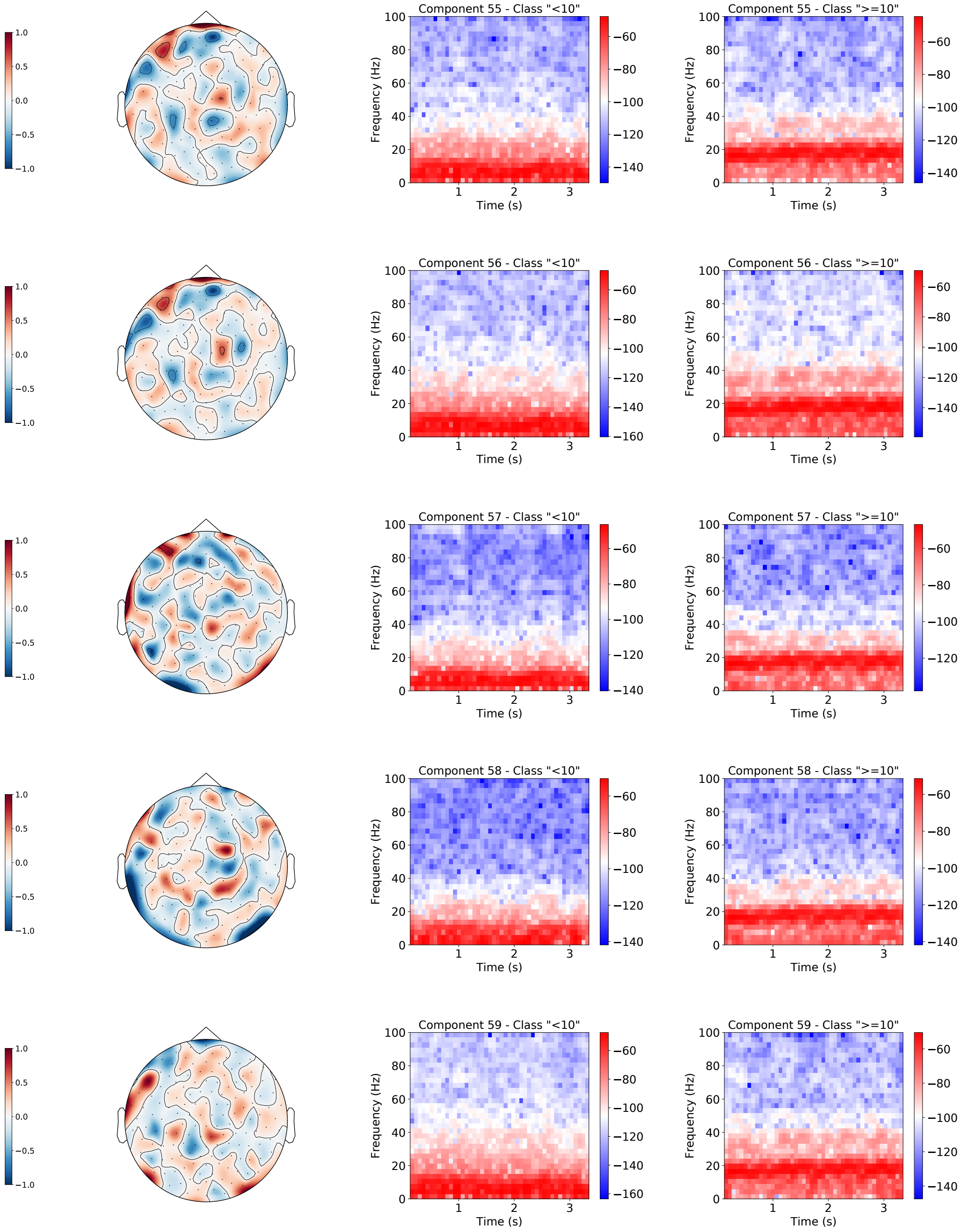
Component 55 - Class "<10"
Component 55 - Class ">=10"
Component 56 - Class "<10"
Component 56 - Class ">=10"
Component 57 - Class "<10"
Component 57 - Class ">=10"
Component 58 - Class "<10"
Component 58 - Class ">=10"
Component 59 - Class "<10"
Component 59 - Class ">=10"
Frequency (Hz)
Time (s)
